# Retraction: The Circular RNA Cdr1as Act as an Oncogene in Hepatocellular Carcinoma through Targeting miR-7 Expression

**DOI:** 10.1371/journal.pone.0283112

**Published:** 2023-03-29

**Authors:** The PLOS ONE Editors

The *PLOS ONE* Editors retract this article [1] because it was identified as one of a series of submissions for which we have concerns about peer review, authorship, and similarities across articles. These concerns call into question the validity and provenance of the reported results. We regret that the issues were not addressed prior to the article’s publication.

LY, XG, LS, QZ, and BL either did not respond directly or could not be reached. LZ responded but expressed neither agreement nor disagreement with the editorial decision.

Fig 5D appears to report material similar to that reported in [2], published in 2015, John Wiley & Sons Ltd., and the shCDR1as panel of Fig 2F and the miR-7 mimic panel of Fig 3E appear to report material similar to that reported in [3,4], published in 2015, Elsevier B.V., which are not offered under a CC-BY license and are therefore excluded from this article’s [1] license. At the time of retraction, the article [1] was republished to note this exclusion in the legend of Figs 2, 3, and 5, and the article’s copyright statement.
